# Advances in Electrochemical Biosensor Technologies for the Detection of Nucleic Acid Breast Cancer Biomarkers

**DOI:** 10.3390/s23084128

**Published:** 2023-04-20

**Authors:** Ana-Maria Chiorcea-Paquim

**Affiliations:** 1 University of Coimbra, CEMMPRE, ARISE, Department of Chemistry, 3004-535 Coimbra, Portugal; anachior@ipn.pt; 2 Instituto Pedro Nunes, 3030-199 Coimbra, Portugal

**Keywords:** cancer biomarker, breast cancer, microRNA, mi-RNA, miR-21, miR-155, BRCA1, electrochemistry, electrochemical biosensor

## Abstract

Breast cancer is the second leading cause of cancer deaths in women worldwide; therefore, there is an increased need for the discovery, development, optimization, and quantification of diagnostic biomarkers that can improve the disease diagnosis, prognosis, and therapeutic outcome. Circulating cell-free nucleic acids biomarkers such as microRNAs (miRNAs) and breast cancer susceptibility gene 1 (BRCA1) allow the characterization of the genetic features and screening breast cancer patients. Electrochemical biosensors offer excellent platforms for the detection of breast cancer biomarkers due to their high sensitivity and selectivity, low cost, use of small analyte volumes, and easy miniaturization. In this context, this article provides an exhaustive review concerning the electrochemical methods of characterization and quantification of different miRNAs and BRCA1 breast cancer biomarkers using electrochemical DNA biosensors based on the detection of hybridization events between a DNA or peptide nucleic acid probe and the target nucleic acid sequence. The fabrication approaches, the biosensors architectures, the signal amplification strategies, the detection techniques, and the key performance parameters, such as the linearity range and the limit of detection, were discussed.

## 1. Introduction

**Figure 1:**
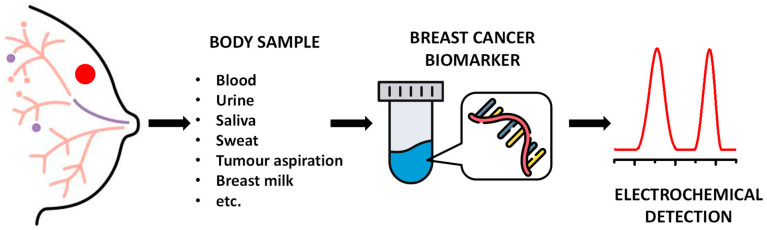
Schematic representation of the electrochemical detection of nucleic acid breast cancer biomarkers that can be obtained via body samples.

Breast cancer remains one of the main causes of mortality in women; its occurrence increases with age, especially after the age of 65 [1,2]. The increased need for breast cancer early detection requires the discovery, development, optimization, and efficient analytical detection of diagnostic biomarkers that can improve the disease prognosis and the therapeutic outcome [3]. Cancer-associated biomarkers are generally nucleic acids (DNA and RNA sequences) [4,5,6,7,8,9,10], proteins [11,12], exosomes [13,14], or whole cells [15]. Among them, circulating nucleic acids may reflect the characteristics of the primary tumor cells and the micrometastatic cells, and, therefore, are considered excellent biomarkers for screening breast cancer in biological fluids [16], as shown in Figure 1.

Noncoding RNAs (ncRNAs) are a family of functional RNA molecules without the protein-coding feature that represents an important part of the human transcriptome, e.g., ribosomal RNAs, transfer RNAs, circular RNAs, long ncRNAs, and short ncRNAs. Among short ncRNAs, microRNAs (miRNAs) are a highly conserved family of transcripts with lengths of approximately 20–25 nucleotides, which primarily regulate gene expression by promoting messenger RNA degradation or repressing its translation [17]. miRNAs are regulators of a variety of cellular processes involving development, differentiation, and signaling; their aberrant expression is associated with a variety of human diseases, including cancer and immune dysfunctions. miRNAs received increased attention as cancer biomarkers for the non-invasive early diagnosis, detection, and treatment of breast cancer because they are soluble and observable in cancer cells, blood, plasma, and patients’ saliva [3]. Moreover, their large amount in biological fluids allows for an easy detection without sample treatment. The most known miRNAs biomarkers used in breast cancer research are miR-21, miR-155, miR-222, and miR-1246, while a number of other miRNAs, such as miR-9, miR-10a, miR-10b, miR-93, miR-125b, miR-181d-5p, miR-191, miR-200, miR-205, miR-221/222, miR-374a, miR-375, miR-378e, and let-7, are implicated in various stages of breast cancer metastasis [16]. Moreover, miR-7, miR-29b, miR-34a, miR-124, miR-153, miR-141, miR-148a, miR-152-3p, miR-205, and miR-497 are known to act as tumor suppressors in breast cancer.

Breast cancer susceptibility genes 1 and 2 (BRCA1 and BRCA2) are antioncogenes expressed in the breast and ovarian cells of women genetically predisposed to breast and ovarian cancer, where they play an important role in the repair of the chromosomal damage. Mutations in the BRCA1 lead to malfunctions and increase the risk of breast cancer. Therefore, the detection of BRCA1 biomarker allows for the characterization of the genetic features and the screening of breast or ovarian cancer patients.

The usual methods for the analysis of nucleic acid breast cancer biomarkers are based on traditional molecular biology techniques, such as cloning, enzymatic ligation assays, Northern blot analysis, oligonucleotide microarray methods, quantitative real-time polymerase chain reaction (qRT-PCR), in situ hybridization, and deep sequencing [18,19], but they require many experimental steps, are expensive, laborious, time consuming, and require highly trained biologists.

Electrochemical methods, including cyclic voltammetry (CV), differential pulse voltammetry (DPV), square wave voltammetry (SWV), amperometry (A), and electrochemical impedance spectroscopy (EIS) are highly sensitive and selective in detecting different analytes [20,21,22,23,24,25], offering excellent platforms for the detection of breast cancer biomarkers. Electrochemical biosensors have seen promising developments in the field of clinical diagnosis due to their robustness, easy miniaturization, low detection limits, low cost, use of small analyte volumes, and the ability to be used in turbid biofluids with optically absorbing and fluorescing compounds when compared with optical, magnetic, and colorimetric biosensors [26]. In particular, electrochemical DNA biosensors have received increased attention due to their ability to easily detect biomarkers of DNA oxidative damage [27,28,29,30] and of disease [31,32,33].

According to IUPAC, an electrochemical DNA biosensor is a device consisting of an electrochemical transducer (the electrode) with a biological recognition element (the DNA film) immobilized on its surface [34]. Its applications are generally based on detecting the interaction between the analyte under investigation and the immobilized DNA [4,5,6,7,8,9,10]. Using this approach, electrochemical DNA biosensors have been successfully used to study DNA interactions with target nucleic acid sequences [31,32,33], pharmaceutical drugs [9,10,35,36,37], proteins [38,39], hazard carcinogenic compounds [40], and pollutants [9].

This review presents the recent advances on the electroanalytical methods of detection and quantification of miRNAs and BRCA1 breast cancer biomarkers using electrochemical DNA biosensors, consisting on the detection of hybridization events between a DNA or PNA (peptide nucleic acid) capture probe and the target nucleic acid sequence. The biosensors fabrication strategies, based on functional nanomaterials, oligonucleotides, and enzymes, the detection techniques, and the key performance parameters, such as the linearity range and limit of detection (LOD), are discussed.

## 2. Electrochemical Biosensors for miRNA Analysis

In recent years, various approaches for the development of electrochemical biosensors for miRNA analysis have been reported [32,41,42], as shown in Table 1. Generally, they are constructed by immobilizing short, complementary single-stranded DNA (ssDNA) capture probes onto the surface of electrochemical transducers; the hybridization with the target miRNA is measured either directly (label-free electrochemical biosensors) or via changes of the redox signal of an electroactive label.

**Figure 2:**
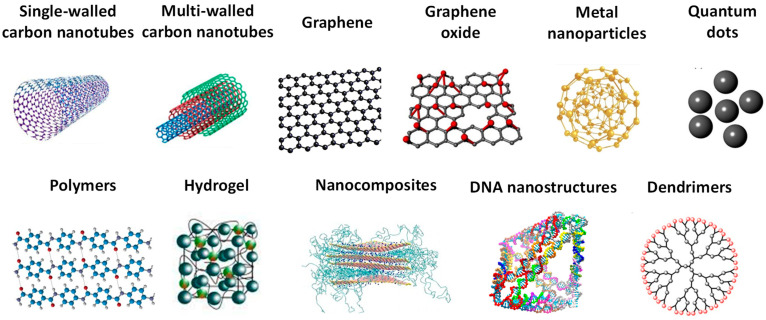
Schematic representation of the nanostructured materials usually employed in the fabrication of electrochemical biosensors for the detection of nucleic acid breast cancer biomarkers.

The biosensor electrochemical performance is closely related with its interfacial structure, which profoundly affects both the thermodynamics and kinetics of the DNA-miRNA binding and the signal transduction of the biomolecules. Therefore, to enhance the electron transfer, to improve the biosensor capture efficiency, and to amplify the electrochemical signal, various integrated nanostructured materials have been employed [19,32], as shown in Figure 2. Examples may include metal nanoparticles (NPs) [43,44,45,46,47,48,49,50], nanorods (NRs) [51,52], nanostructures [53,54,55], graphene (G), graphene oxide (GO) [51,56], reduced graphene oxide (rGO) [54,57], graphene quantum dots (GQDs) [54,58], carbon nanotubes (CNs) [57,59], polymers [49,60,61], hydrogels [61,62], DNA nanostructures [63,64], and dendrimers [59].

**Table 1:** Selected studies concerning the electroanalytical determination of nucleic acid breast cancer biomarkers in standard solutions containing only the analyte *.

Biomarker Analyte	Biosensor Design	Redox Probe	Technique	Linear Range	LOD	Ref.
**miRNA**						
miR-21	ITO/PET/hydrogel-ssDNA	Fc	DPV	1.0 × 10^−8^–5.0 × 10^−5^ M	5.0 × 10^−9^ M	[62]
	SPCE/rGOs/Au NPs/SH-ssDNA; Fc-Au NPs-ssDNA	Fc	DPV	1.0 × 10^−14^–2.0 × 10^−12^ M	5.0 × 10^−15^ M	[65]
	Au/PNA21, PNA155; Fc-CHA21, Mb-CHA155 detection	Fc	SWV	1.0 × 10^−14^–5.0 × 10^−9^ M	2.4 × 10^−15^ M	[66]
	PGE/CB-Au NPs/ssDNA	Mb	DPV	2.9 × 10^−15^–7.0 × 10^−7^ M	1.0 × 10^−15^ M	[46]
	Au/chitosan/ssDNA origami	Mb	DPV	1.0 × 10^−13^–1.0 × 10^−8^ M	8.0 × 10^−14^ M	[64]
	Au/LNA-TWJ	Mb, TCEP	ACV	1.0 × 10^−16^–1.0 × 10^−10^ M	7.7 × 10^−17^ M	[67]
	PGE/PPy/ssDNA	MDB	DPV	–––	1.7 × 10^−10^ M	[47]
	Au/Au NPs-PPy/ssDNA	TB	DPV	1.0 × 10^−16^–1.0 × 10^−9^ M	7.8 × 10^−17^ M	[68]
	SPCE/Au NPs/ssDNA	K_3_[Fe(CN)_6_], [Ru(NH_3_)_6_]Cl_3_	SWV	1.0 × 10^−15^–1.0 × 10^−11^ M	4.0 × 10^−16^ M	[69]
	GCE/Au NPs/DNA	Ag NPs	LSV	1.0 × 10^−16^–5.0 × 10^−14^ M.	2.0 × 10^−17^ M	[70]
	GCE/SA/ssDNA	[Fe(II)(CN)_6_]^4−^/Fe(III)(CN)_6_]^3−^	EIS	1.0 × 10^−14^–1.0 × 10^−8^ M	2.0 × 10^−14^ M	[71]
	GCE/HP1, HP2, DG-TIS	[Fe(II)(CN)_6_]^4−^/Fe(III)(CN)_6_]^3−^	DPV	5.0 × 10^−14^–5.0 × 10^−7^ M	3.5 × 10^−14^ M	[63]
	SPCE/Au NPs/ssDNA	HRP	SWV	1.9 × 10^−5^–1.0 × 10^−1^ M	1.9 × 10^−14^ M	[72]
	μPAD/Au NRs	CeO_2_-Au@GOx	DPV	1.0 × 10^−15^–1.0 × 10^−12^ M	4.3 × 10^−16^ M	[52]
	GCE/MoS_2_-Thi-Au NPs/ssDNA	MoS2-Thi-Au NPs	SWV	1.0 × 10^−12^–1.0 × 10^−8^ M	2.6 × 10^−13^ M	[73]
	Au/SWCNs/NDs/ssDNA-HCR-hemin/GQ DNAzyme	–––	DPV	1.0 × 10^−14^–1.0 × 10^−9^ M	2.0 × 10^−15^ M	[48]
	GCE/rGO/β-CD/HP-DNAzyme	Fc	DPV	1.0 × 10^−15^−1.0 × 10^−10^ M	1.8 × 10^−15^ M	[74]
	Au/HCP1-HCP2, ssDNA1/Fe_3_O_4_ NPs/Thi, ssDNA2/Fe_3_O_4_ NPs/Fc, HCR	Thi, Fc	DPV	––	4.6 × 10^−16^ M	[75]
miR-155	CPE/Fe_3_O_4_NPs@Ag/NH_2_-ssDNA	RSV	DPV	5.0 × 10^−16^ –1.0 × 10^−9^ g/mL	1.5 × 10^−16^ g/mL	[45]
	GCE/GO/Au NRs/SH-ssDNA	OB	DPV	2.0 × 10^−15^–8.0 × 10^−12^ M	6.0 × 10^−14^ M	[51]
	GCE/Fe-Ni@rGO/QD-Ag, Au NS/SH-ssDNA	hematoxylin	DPV	5.0 × 10^−20^–5.0 × 10^−11^ M	2.0 × 10^−17^ M	[54]
	GCE/MWCNTs/PtNPs/DNA, CHA, PSC@Au NPs-ALP, NPP	PMo_12_O_40_^3−^	DPV	1.0 × 10^−14^–1.0 × 10^−9^ M	1.6 × 10^−15^ M	[44]
	Au/PNA21, PNA155—CHA	Mb	SWV	5.0 × 10^−14^–5.0 × 10^−8^ M	1.1 × 10^−14^ M	[66]
	Au/ssDNA-GQDs	HRP	A	1.0 × 10^−15^–1.0 × 10^−13^ M	1.4 × 10^−16^ M	[58]
	GCE; LCR, MB–CP1CP2; PbS-QDs, CdS-QDs	PbS-QDs, CdS-QDs	SWV	5.0 × 10^−14^–3.0 × 10^−11^ M	1.2 × 10^−14^ M	[76]
miR-24	GCE/MWCNT-PAMAM/ssDNA	Mb	DPV	1.0 × 10^−14^–1.0 × 10^−7^ M	5.0 × 10^−16^ M	[59]
	GCE/PANI-PA/ssDNA	–––	DPV	1.0 × 10^−15^–1.0 × 10^−12^ M	3.4 × 10^−16^ M	[61]
miR-122	Au/Au NPs/rGO/SH-ssDNA	[Fe(II)(CN)_6_]^4−^/Fe(III)(CN)_6_]^3−^	DPV	1.0 × 10^−11^–1.0 × 10^−5^ M	1.7 × 10^−12^ M	[77]
	SPGE/ssDNA	–––	DPV	–––	5.0 × 10^−9^ M	[78]
miR-34a	PGE/ssDNA	[Co(phen)_3_^3+^]	DPV	1.4 × 10^−7^–4.3 × 10^−7^ M	8.4 × 10^−8^ M	[79]
	CA-IL-PGE/ssDNA	–––	DPV	2.0 × 10^−3^–10 × 10^−2^ g L^−1^	1.3 × 10^−7^ M (8.8 × 10^−4^ g L^−1^)	[80]
	GO-PGE/ssDNA	–––	DPV	1.0 × 10^−2^–4.0 × 10^−2^ g L^−1^	7.0 × 10^−7^ (5.0 × 10^−3^ g L^−1^)	[56]
miR-522	HMDE/ssDNA-MB	Os(VI)bipy	DPV	1.0 × 10^−8^–2.0 × 10^−7^ M	––	[81]
	HDME/ssDNA	Os(VI)bipy	DPV	2.0 × 10^−9^–4.0 × 10^−8^ M	2.0 × 10^−9^ M	[82]
miR-141	Au/rGE/CNTs/ssDNA; ELISA-like amplification	HRP	SWV	1.0 × 10^−14^–1.0 × 10^−9^ M	1.0 × 10^−14^ M	[57]
	GCE/poly(JUGco-JUGA)	–––	SWV	5.0 × 10^−13^–1.0 × 10^−10^ M	6.5 × 10^−13^ M	[60]
	Au/HCP1-HCP2, ssDNA1/Fe_3_O_4_ NPs/Thi, ssDNA2/Fe_3_O_4_ NPs/Fc, HCR	Thi, Fc	DPV	––	4.4 × 10^−16^ M	[75]
miR-27b	GCE; LCR, MB–CP1CP2; PbS-QDs, CdS-QDs	PbS-QDs, CdS-QDs	SWV	5.0 × 10^−14^ –1.1 × 10^−9^ M	3.1 × 10^−14^ M	[76]
miR-103	GCE/Au NPs/JUGMHA/NH_2_-ssDNA		SWV	1.0 × 10^−8^–5 × 10^−9^ M	1.0 × 10^−13^ M	[49]
let-7	Au/GQ-DNA-CHA-hemin/GQ DNAzyme	–––	DPV	1.0 × 10^−15^–1.0 × 10^−9^ M	4.6 × 10^−16^ M	[83]
**BRCA1**						
BRCA1	Au/MCH/PNA	Fc-PBA	SWV	1.0 × 10^−14^–1.0 × 10^−8^ M	2.9 × 10^−15^ M	[84]
	GCE/PEDOT/PEP	MB	DPV	1.0 × 10^−14^–1.0 × 10^−9^ M	3.4 × 10^−15^ M	[85]
	Au/Cys/Glu/Fc-PAMAMs/ssDNA;	Fc-PAMAM	DPV	1.3 × 10^−9^–2.0 × 10^−8^ M	4.0 × 10^−10^ M	[86]
	GCE/GO-CB [7]; Fc Au NS/T-DNA/HRP-Au NSs	HRP	DPV	1.0 × 10^−7^–5.0 × 10^−11^ M	2.5 × 10^−11^ M	[87]
	GCE/P[(DA-β-CD)/CTAB-Ag NPs]/MCM−41-SO_3_H	HRP	SWV	6.3 × 10^−10^–2.0 × 10^−9^ g L^−1^	––	[88]
	GCE/P[(DA-β-CD)/CTAB-Ag NPs]/MCM-41-SO_3_H	HRP	DPV	1.6 × 10^−11^–1.0 × 10^−8^ g L^−1^	––	[88]
	Au/cDNA/MCH/CESA/3-QD@DNA NC	–––	DPV	5.0 × 10^−18^–5.0 × 10^−15^ M	1.2 × 10^−18^ M	[89]
	SPE/TDNA/BSA/polyA Au NPs-ssDNA;Biotin-BRCA1/SA-HPR; SH-TMB/H_2_O_2_	–––	CV; A	1.0 × 10^−15^–1.0 × 10^−9^ M	1.0 × 10^−16^ M	[90]
	GCE/rGO/MWCNTs/PANHS/ssDNA	–––	EIS	1.0 × 10^−18^–1.0 × 10^−10^ mol L^−1^	3.5 × 10^−19^ mol L^−1^	[91]
	GCE/MWCNTs/PANHS/ssDNA	–––	EIS	1.0 × 10^−17^–1.0 × 10^−10^ mol L^−1^	3.1 × 10^−18^ mol L^−1^	[91]
	GCE//PANHS/ssDNA	–––	EIS	1.0 × 10^−16^–1.0 × 10^−10^ mol L^−1^	3.7 × 10^−17^ mol L^−1^	[91]
	GCE/PEG/ssDNA	–––	EIS	5.0 × 10^−14^–1.0 × 10^−9^ M	1.7 × 10^−15^ M	[92]
	ITO/CHIT-co-PANI/ssDNA	–––	EIS	5.0 × 10^−17^–2.5 × 10^−14^ M	5.0 × 10^− 17^ M	[93]
	Au/SH-ssDNA	–––	EIS	1.0 × 10^−19^–1.0 × 10^−7^ M	4.6 × 10^−20^ M	[94]
	GCE/PEG/Fe(III)-TA)/pDA; Au NPs	–––	EIS	1.0 × 10^−16^–1.0 × 10^−11^ M	5.0 × 10^−17^ M	[95]

* Abbreviations: 1-naphthyl phosphate (NPP); 1-pyrenebutyric acid-N-hydroxysuccinimide ester (PANHS); 3,3′,5,5′-tetramethylbenzidine (TMB); 5-hydroxy-3-hexanedithiol-1,4-naphthoquinone (JUG-SH), 6-mercaptohexanoic acid (6-MHA); 6-Mercaptohexanol (MCH); alkaline phosphatase (ALP); alternating current voltammetry (ACV); amino-modified ssDNA (NH_2_-ssDNA); amperometry (A); bovine serum albumin (BSA); capture probe (CP); carbon black (CB); carbon nanotubes (CNTs); carbon paste electrode (CPE); catalyzed hairpin assembly (CHA); chemically activated (CA); chitosan (CHIT); cobalt phenanthroline ([Co(phen)_3_^3+^]); cucurbit[7]uril (CB [7]); cyclic enzymatic signal amplification (CESA); cyclic voltammetry (CV); cysteamine (Cys); differential pulse voltammetry (DPV); digestion-to-growth regulated tandem isothermal amplification (DG-TIS); double-strand specific nuclease (DSN) and triple-CdTe QD-labeled DNA NC (3-QD@DNA NC); electrochemical impedance spectroscopy (EIS); ferrocene-labeled ssDNA detection probe (Fc-ssDNAdp); ferrocene (Fc); glassy carbon electrode (GCE); glucose oxidase (GOx); glutaraldehyde (Glu); gold nanoparticles-modified polystyrene magnetic microspheres (PSC@Au NPs); G-quadruplex (GQ); graphene (G); graphene oxide (GO); graphene quantum dots (GQDs); hairpin capture probe (HCP); hairpin probe (HP); hanging mercury drop electrode (HMDE); horseradish peroxidase (HRP); horseradish peroxidase (HRP); hybridization chain reaction (HCR); indium tin oxide/polyethylene terephthalate (ITO/PET); indium-tin-oxide (ITO); ionic liquid (IL); linear-sweep voltammetry (LSV); locked nucleic acid (LNA); magnetic beads (MBs); Meldola’s blue (MDB); mercaptoacetic acid (MCH); methylene blue (Mb); microfluidic paper-based analytical device (μPAD); mobile crystalline material-41 grafted by sulfonic acid (MCM-41-SO_3_H); molybdophosphate anion (PMo_12_O_40_^3−^); multiwalled carbon nano-tubes (MWCNTs); nanocomposites (NCs); nanodiamonds (NDs); nanoparticles (NPs); nanorods (NRs); nanosphere (NS); nanostars (NSs); Oracet Blue (OB); pencil graphite electrode (PGE); peptide nucleic acid (PNA); phenylboronic acid (PBA); phytic acid (PA); poly(5-hydroxy-1,4-naphthoquinone-co-5-hydroxy-2-carboxyethyl-1,4-naphthoquinone) (poly(JUGco-JUGA)); poly(amidoamine) (PAMAM); poly-adenine (polyA); polyaniline (PANI); poly-dopamine (pDA); polyethylene glycol (PEG); polypyrrole (PPy); polyvinylpyrrolidone (PVP); quantum dot (QD); reduced graphene oxide (rGO); resveratrol (RSV); screen-printed graphite electrode (SPGE); silver-conjugated graphene quantum dots (GQD-Ag); single-stranded DNA (ssDNA); single-walled carbon nanotubes (SWCNs); six-valent osmium and 2,2′-bipyridine (Os(VI)bipy); square wave voltammetry (SWV); streptavidin (SA); sulfonic acid (SA); tannic acid (TA); tetrahedral-DNA (TDNA); thiolated ssDNA (SH-ssDNA); thiolated ssDNA capture probe (SH-ssDNAcp); thionine (Thi); three-way junction (TWJ); toluidine blue (TB); tris (2- carboxyethyl) phosphine (TCEP); β-cyclodextrin (β-CD).

### 2.1. Electrochemical Biosensors Based on Redox Mediators

Many electrochemical biosensors for miRNA detection are based on the detection of redox mediators that can be either attached to the DNA probe or the sensing assembly, or produced within the biosensing system. The electronic mediators can be (i) redox molecules, e.g., ferrocene (Fc) [62,66,74], methylene blue (Mb) [46,64,66], Oracet Blue (OB) [51,96], resveratrol (RSV) [45], ruthenium oxide [97], (ii) redox couples, e.g., [Fe(III)(CN)_6_]^3−^/[Fe(II)(CN)_6_]^4−^ [71], (iii) redox enzymes [98], or (iv) polymeric systems [49,60,61]. When the hybridization of the DNA probe with the target miRNA takes place, the redox probe electrochemical signal varies proportionally to the miRNA concentration.

#### 2.1.1. Biosensor Based on Redox Molecules

miR-21 and miR-155 have been demonstrated to play a major role in breast cancer progression, which makes them especially important in breast cancer detection [16].

An electrochemical biosensing platform for miR-21 based on the Fc-tagged DNA hydrogel self-assembled onto indium tin oxide (ITO)/polyethylene terephthalate (PET) electrode was proposed [62], which showed a linear range from 1.0 × 10^−8^ M to 5.0 × 10^−5^ M and an LOD of 5.0 × 10^−9^ M.

**Figure 3:**
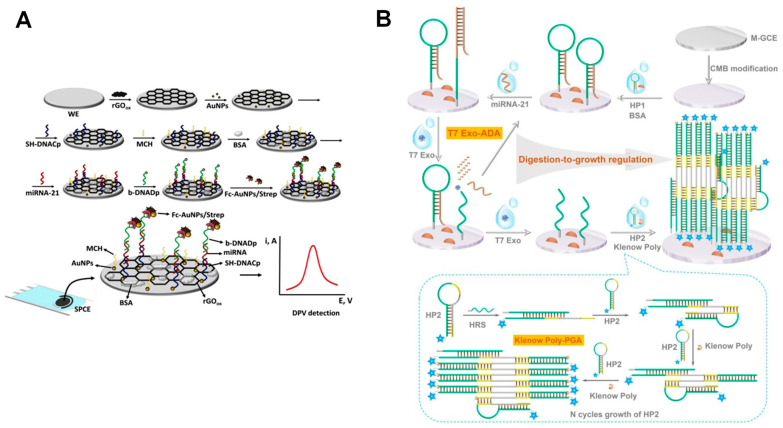
Schematic representation of different strategies employed for electrochemical biosensors for miR-21 detection, based on (**A**) Fc-Au NPs redox label, and (**B**) [Fe(II)(CN)_6_]^4−^/Fe(III)(CN)_6_]^3−^ redox couple and DG-TIS amplification. Reproduced from [63,65] with permission.

In a different report, a disposable biosensor for the voltammetric determination of miR-21 was reported [65], consisting of a sandwich-type hybridization architecture, which used two DNA probes (one for capture and the other one for detection) that hybridized contiguously with the target miRNA-21, as shown in Figure 3A. The first thiolated ssDNA (SH-ssDNA) capture probe was immobilized onto screen-printed carbon electrodes (SPCEs) modified by rGO and Au NPs, while the second Fc-Au NPs-labeled ssDNA detection probe was used as a carrier for the redox probe. Based on this strategy, the biosensor showed a linear range from 1.0 × 10^−14^ to 2.0 × 10^−12^ M and an LOD of 5.0 × 10^−15^ M.

An electrochemical biosensor for miR-21, consisting of Mb-labeled SH-ssDNA probes immobilized onto carbon black (CB) and gold NPs (Au NPs)-modified pencil graphite electrodes (PGEs), was also reported [46]. After hybridization with the target miR-21, the orientation of the labeled capture probe changed, which caused a decrease in the DPV response of the Mb oxidation peak current. The sensor showed a linear range from 2.9 × 10^−15^ to 7.0 × 10^−7^ M and an LOD of 1.0 × 10^−15^ M. In addition, based on Mb labeling, an amplification-free electrochemical biosensor was developed using DNA origami nanostructured probes self-assembled onto chitosan-modified gold electrodes [64], which showed a linear range from 1.0 × 10^−13^ to 1.0 × 10^−8^ M and an LOD of 8.0 × 10^−14^ M.

A three-way junction (TWJ) DNA electrochemical biosensor for miR-21 detection was also developed [67], using locked nucleic acid (LNA) as the capture probes, immobilized onto gold electrodes, and Mb and tris (2- carboxyethyl) phosphine (TCEP) redox labels. The biosensor showed a linear range from 1.0 × 10^−16^ to 1.0 × 10^−10^ M and an LOD of 7.7 × 10^−17^ M.

miR-21 was also detected at PGEs modified by electropolymerized polypyrrole (PPy) and ssDNA probes labeled with Meldola’s blue (MDB), showing an LOD of 1.7 × 10^−10^ M by DPV [47]. In another report, Au NPs and PPy superlattices were used to amplify the electrochemical signal of the toluidine blue (TB) label [68], with the Au/Au NPs-PPy/ssDNA biosensor showing a linear range from 1.0 × 10^−16^ to 1.0 × 10^−9^ M and an LOD of 7.8 × 10^−17^ M.

Using K_3_[Fe(CN)_6_] and [Ru(NH_3_)_6_]Cl_3_ redox probes, an electrochemical biosensor which enabled three detection modalities based on miR-21 hybridization, p19 protein binding, and protein displacement was also reported [69]. The sensor achieved the miR-21 detection by SWV via a hybridization protocol with a complementary SH-ssDNA probe self-assembled onto Au NPs-modified SPCEs in the linear range from 1.0 × 10^−15^ to 1.0 × 10^−11^ M with an LOD of 4.0 × 10^−16^ M.

An electrochemical biosensor for miR-21 was also developed [70], based on hairpin-like DNA probes immobilized onto a Au NPs-modified glassy carbon electrode (GCE) and the in situ formation of Ag NPs aggregate labels, detected by linear-sweep voltammetry (LSV). The sensor showed an LOD of 2.0 × 10^−17^ M in the linear range from 1.0 × 10^−16^ to 5.0 × 10^−14^ M.

Using a ferricyanide/ferrocyanide, [Fe(II)(CN)_6_]^4−^/Fe(III)(CN)_6_]^3−^, redox couple, miR-21 was specifically detected at an electrochemical biosensor consisting of ssDNA capture probes immobilized onto a GCE modified by sulfonic acid deposition with subsequent chlorination [71]. The sensor presented a linear range from 1.0 × 10^−14^ to 1.0 × 10^−8^ M and an LOD of 2.0 × 10^−14^ M.

Furthermore, based on the [Fe(II)(CN)_6_]^4−^/Fe(III)(CN)_6_]^3−^ redox couple, an electrochemical biosensor for miRNA-21 detection was developed [63], which used a digestion-to-growth regulated tandem isothermal amplification (DG-TIS) and two hairpin probes as the enzymatic reaction units, as shown in Figure 3B. The sensor showed a linear range from 5.0 × 10^−14^ to 5.0 × 10^−7^ M and an LOD of 3.5 × 10^−14^ M, and was able to easily distinguish between one-base mismatched sequences.

miR-155 is a particularly important biomarker for urine sample analyses since increased levels of miR-155 in the urine of breast cancer patients were observed, when compared with the miR-21 lower expression in urine, when compared with healthy controls [16].

**Figure 4:**
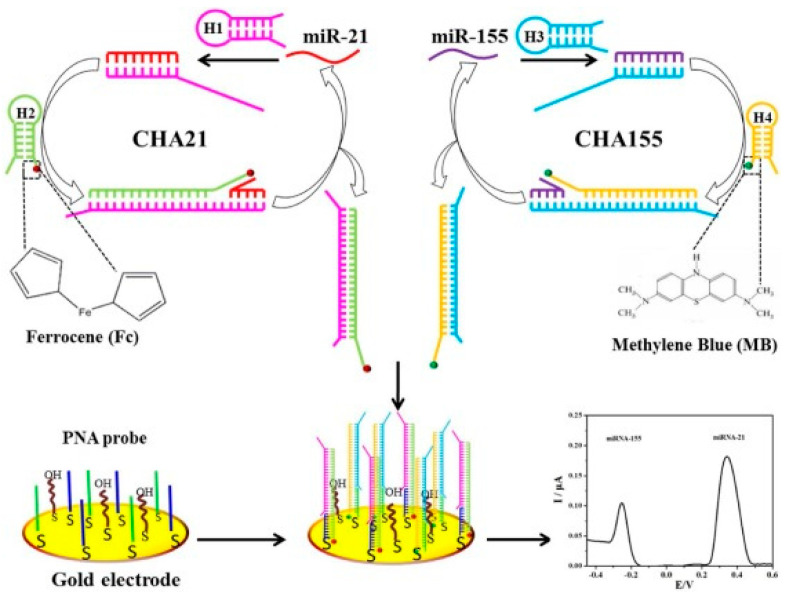
Schematic representation of the electrochemical biosensor for simultaneous detection of miR-21 and miR-155, based on coupling the PNA immobilization with target-triggered CHA amplifications. Reproduced from [66] with permission.

An electrochemical method for the simultaneous detection of miR-21 and miR-155 used a strategy based on 2 redox labels, Fc corresponding to miR-21 and Mb to miR-155 detection, coupled with a target-catalyzed hairpin assembly (CHA), as shown in Figure 4 [66]. Gold electrodes were modified by two peptide nucleic acid (PNA) probes, one for each miRNA target; in the presence of miR-21 and miR-155, the CHA was triggered selectively between the two hairpins, one labeled with Fc and the other with Mb. The resulting redox label-modified CHA products (Fc-CHA21 or Mb-CHA155) were specifically captured by the immobilized PNA probes, with the Fc and Mb labels being detected by SWV. This assay was highly selective for discriminating between the two miRNAs with similar sequences, presenting LODs of 2.4 × 10^−15^ M for miR-21 and 1.1 × 10^−14^ M for miR-155, in the linear ranges from 1.0 × 10^−14^ to 5.0 × 10^−9^ M for miR-21 and from 5.0 × 10^−14^ to 5.0 × 10^−8^ M for mi-155. The method was applied for the determination of miR-21 and miR-155 in human cancer cells.

Based on RSV redox labels, an electrochemical biosensor for the detection of miR-155 was developed [45], consisting of complementary amino-modified ssDNA (NH_2_-ssDNA) probes immobilized onto a magnetic bar carbon paste electrode (CPE) modified by Fe_3_O_4_NPs@Ag core-shell NPs. The hybridization with the target miR-155 was detected by DPV by monitoring the RSV oxidation peaks. The CPE/Fe_3_O_4_NPs@Ag/NH_2_-ssDNA biosensor presented a linear range from 5.0 × 10^−16^ to 1.0 × 10^−9^ M and an LOD of 1.5 × 10^−16^ M. The miR-155 detection without significant interference was successfully achieved in spiked real samples of human serum.

**Figure 5:**
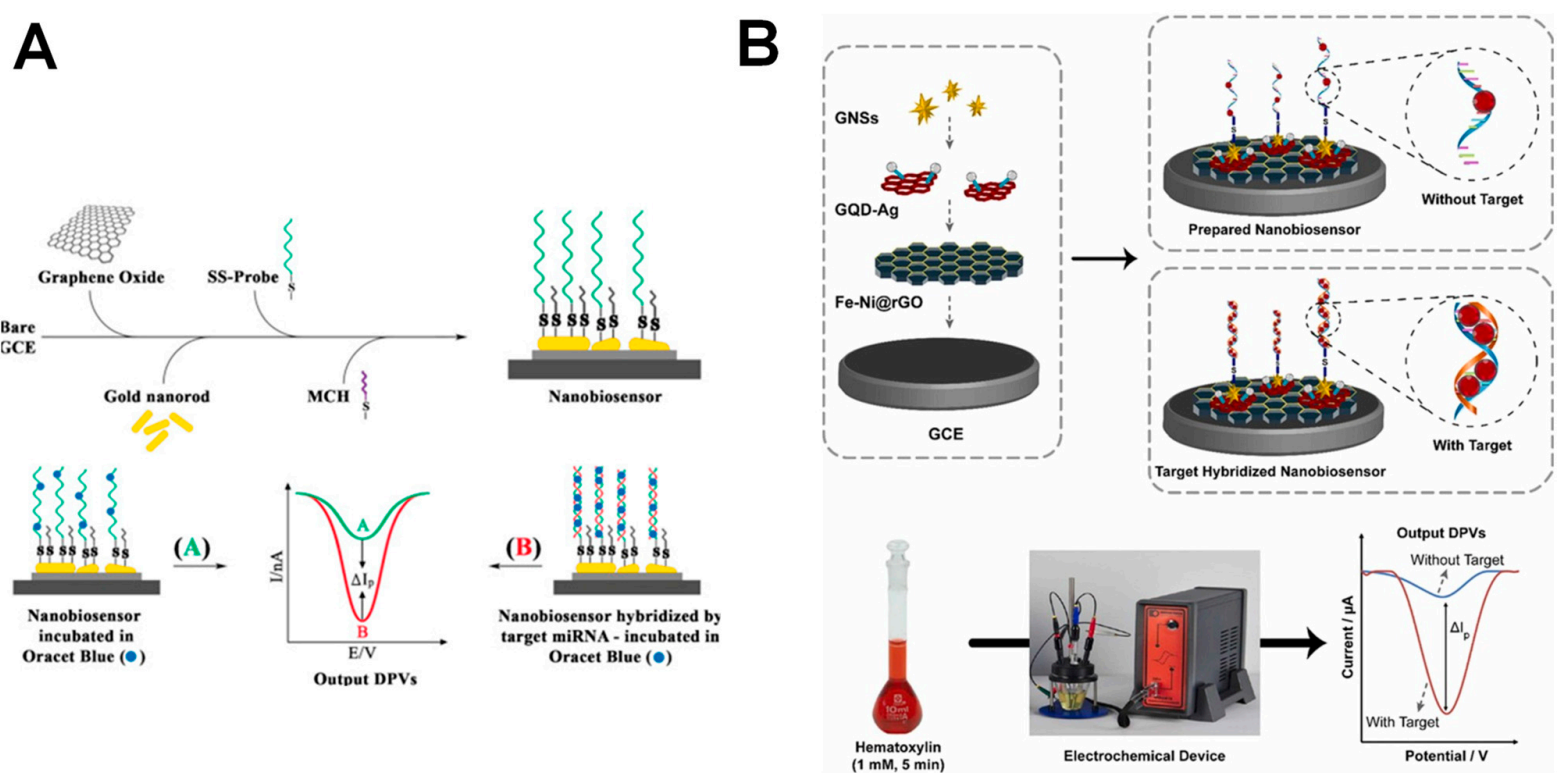
Schematic representation of different strategies employed for electrochemical biosensors for miR-155 detection, based on (**A**) OB (**B**) hematoxylin redox labels. Reproduced from [51,54] with permission.

The electroanalytical detection of miR-155 was achieved via an OB redox label, at an electrochemical biosensor build by immobilizing SH-ssDNA probes onto a GCE modified by GO sheets and Au NRs, as shown in Figure 5A [51]. The hybridization events were detected by DPV, via the OB electrochemical peaks [51,96]. The biosensor presented a linear range from 2.0 × 10^−15^ to 8.0 × 10^−12^ M and an LOD of 6.0 × 10^−14^ M, and was able to discriminate between the target miR-155 and its non-complementary and one- and three-base mismatched sequences.

More recently, a biosensor for miR-155 detection based on a hematoxylin redox label was developed [54]. Its design consisted of SH-ssDNA capture probes immobilized onto a GCE modified by rGO with nickel-iron (Fe-Ni@rGO), silver-conjugated graphene quantum dots (QDs) and Au nanostars (NSs), as shown in Figure 5B. The nanocomposite significantly increased the electrode surface area and conductivity, and enhanced the biosensor sensitivity to an LOD of 2.0 × 10^−17^ M in the linear range from 5.0 × 10^−20^ to 5.0 × 10^−11^ M. The biosensor showed a high specificity for miR-155 detection compared to its non-complementary and one- and three-base mismatched sequences.

**Figure 6:**
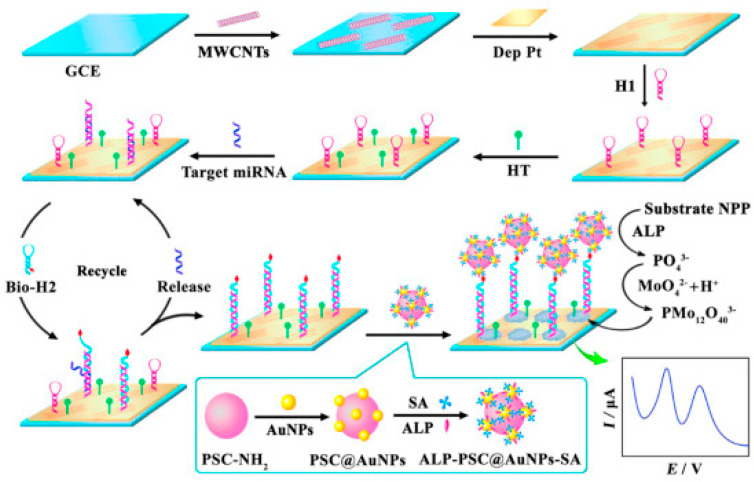
Schematic representation of an electrochemical biosensor for miR-155 detection, based on in situ generation of PMo_12_O_40_^3−^ redox mediator. Reproduced from [44] with permission.

An electrochemical biosensor for miRNA-155 was also developed by coupling a CHA-induced target recycling amplification strategy with the use of an alkaline phosphatase (ALP) enzyme to catalyze the in situ generation of the redox mediator, as shown in Figure 6 [44]. ALP hydrolyzed the substrate 1-naphthyl phosphate (NPP) to produce PO_4_^3−^, which further reacted with acidic molybdate to form molybdophosphate anion (PMo_12_O_40_^3−^). Au NPs-modified polystyrene magnetic microspheres (PSC@Au NPs) were used for immobilizing ALP and streptavidin (SA) to form ALP-PSC@Au NPs-SA bioconjugates. Pt NPs were deposited onto carboxyl-functionalized multiwalled carbon nanotubes (MWCNTs)-modified GCE to capture the hairpin probes. The PMo_12_O_40_^3−^ formed was used as a redox mediator to quantify the target miR-155 in the linear rage from 1.0 × 10^−14^ to 1.0 × 10^−9^ M with an LOD of 1.6 × 10^−15^ M [44].

Along with miR-21 and miR-155, miR-24 is overexpressed in patients with breast cancer, and it is downregulated after surgery or treatment [16].

An electrochemical biosensor for the detection of miR-24, consisting of a GCE modified with MWCNTs and polyamidoamine (PAMAM) dendrimers and immobilized Mb redox labeled ssDNA probes, was described [59]. The biosensor showed a linear range from 1.0 × 10^−14^ to 1.0 × 10^−7^ M and an LOD of 5.0 × 10^−16^ M in the standard samples, and it was also successfully used to detect miR-24 in the total RNA samples extracted from HeLa cells.

A reduced miR-34a expression is strongly associated with tumor progression and indicates a worse prognosis [16]. miR-34a was detected at an electrochemical biosensor based on ssDNA probes labeled with the electroactive metal complex indicator cobalt phenanthroline, [Co(phen)_3_^3+^], immobilized on the surface of a PGE [79]. The proposed sensor showed a linear range from 1.4 × 10^−7^ to 4.3 × 10^−7^ M and an LOD of 8.4 × 10^−8^ M.

A high expression of miR-522 is correlated with poor overall survival in patients with triple-negative breast cancer [99]. In a different report, miR-522 labeled with the electroactive six-valent osmium and 2,2′-bipyridine (Os(VI)bipy) redox probe was hybridized with a complementary biotinylated ssDNA capture probe attached to SA magnetic beads (MBs) [81]. miR-522 was detected at the hanging mercury drop electrode (HMDE) in the linear range from 1.0 × 10^−8^ to 2.0 × 10^−7^ M [81]. Furthermore, relying on the Os(VI)bipy redox label, the MBs-based hybridization between the complementary ssDNA probe and the target miR-522 was detected at HMDE in the linear range from 2.0 × 10^−9^ to 4.0 × 10^−8^ M and an LOD of 2.0 × 10^−9^ M [82].

**Figure 7:**
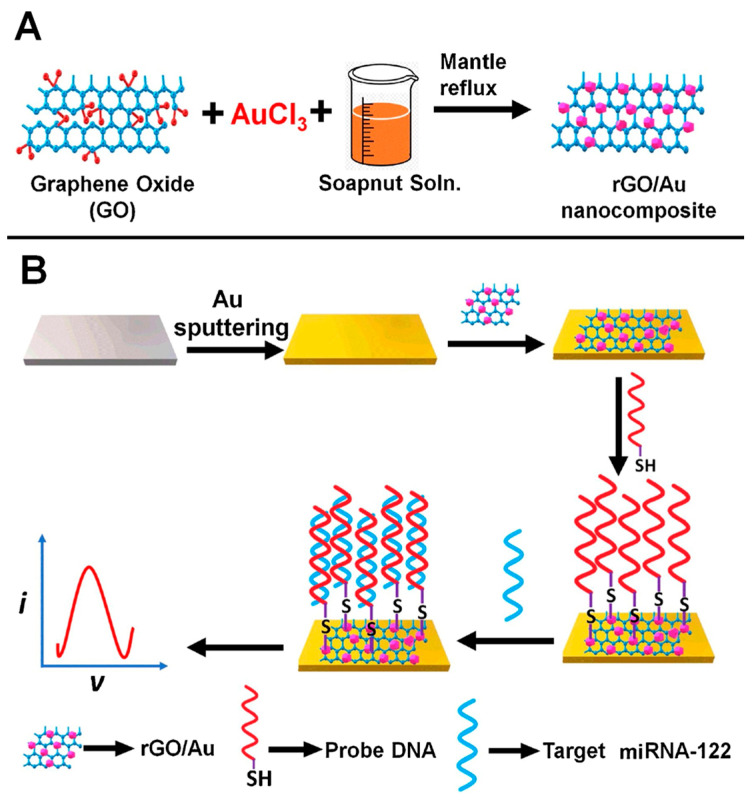
Schematic representation of (**A**) synthesis of rGO/Au nanocomposite and (**B**) rGO/Au nanocomposite-based electrochemical biosensor for miRNA-122 detection. Reproduced from [77] with permission.

High miR-122 levels in the circulation have been associated with metastasis in breast cancer patients [100]. An electrochemical biosensor for the detection of miR-122, based on the electrochemical detection of the [Fe(II)(CN)_6_]^4−^/Fe(III)(CN)_6_]^3−^ redox couple and the immobilization of SH-ssDNA capture probes onto a gold electrode modified by Au NPs and rGO, as shown in Figure 7, showed a linear range from 1.0  × 10^−11^ to 1.0  × 10^−5^ M and an LOD of 1.7  × 10^−12^ M [77].

#### 2.1.2. Biosensors Based on Enzymes

The circulating levels of miR141 play an important role as biomarkers for the early detection of breast cancer metastases [16]. An electrochemical immunosensor for the detection of miR-141 was developed, which used NH_2_-ssDNA probes immobilized onto screen-printed gold electrodes (SPGEs) modified with rGO and carbon nanotubes (CNTs) [57]. The hybridization with the miR-141 target was followed by an ELISA-like amplification strategy via a horseradish peroxidase (HRP) catalytic system: the hydroquinone was oxidized to benzoquinone by the HRP/H_2_O_2_ catalytic system, then the benzoquinone reduction was electrochemically detected, and the catalytic reduction current was correlated with the immobilized HRP [57]. The sensor showed a linear range from 1.0 × 10^−14^ M to 1.0 × 10^−9^ M and an LOD of 1.0 × 10^−14^ M. In a different report, based on DNA tetrahedral probes and enzymatic HRP-assisted amplification, the detection of miR-141 was achieved for concentrations as low as 1.0 × 10^−15^ M [101].

**Figure 8:**
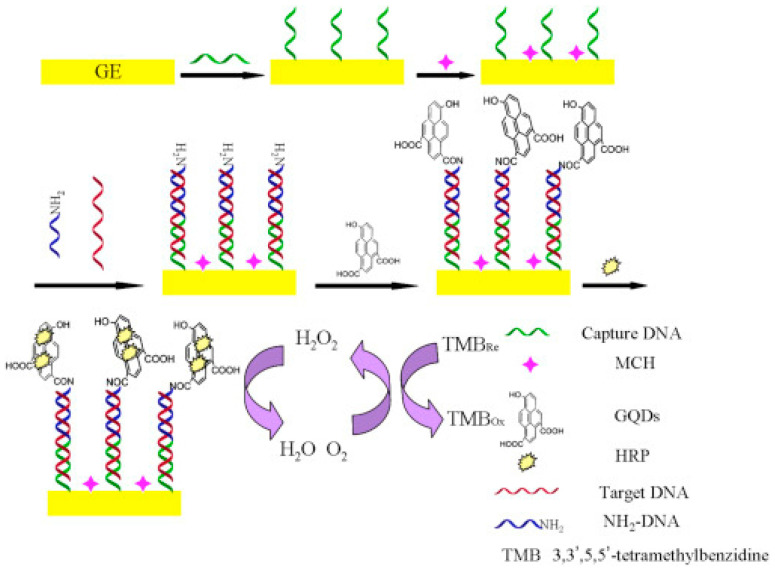
Schematic representation of an enzyme catalytic amplification for miRNA-155 detection at a GQD-based electrochemical biosensor. Reproduced from [58] with permission.

An electrochemical biosensor for miR-155 detection with an HRP enzyme-assisted catalytic reaction and functionalized graphene quantum dots (GQDs) as the sensing element was also developed, as shown Figure 8 [58], showing a linear range from 1.0 × 10^−15^ to 1.0 × 10^−13^ M and an LOD of 1.4 × 10^−16^ M.

Furthermore, based on the HRP which catalyze the oxidation of *o*-phenylenediamine into 2,3-diaminophenazine, the electroanalytical determination of miR-21 was succeeded at an electrochemical biosensor consisting of SH-ssDNA capture probes attached onto Au NPs-modified SPCEs [72]. The sensor showed a linear range from 1.9 × 10^−5^ to 1.0 × 10^−1^ M and an LOD of 1.9 × 10^−14^ M.

#### 2.1.3. Biosensors Based on Electrochemical Indicator–Functionalized Nanomaterials

An electrochemical sensing platform for miR-21 detection was developed using ssDNA probes immobilized onto a GCE modified by MoS_2_ nanosheets functionalized with thionine (Thi) and Au NPs (MoS_2_-Thi-Au NPs) [73]. Upon hybridization, the formation of the probe ssDNA-miR-21 duplex hindered the electron transfer and decreased the Thi electrochemical signal. The sensor showed a limit of detection from 1.0 × 10^−12^ to 1.0 × 10^−8^ M and an LOD of 2.6 × 10^−13^ M.

In a different strategy, an electrochemical biosensor for miR-21 detection consisted of Au NRs modified with a microfluidic paper-based analytical device (μPAD), built using CeO_2_-Au modified by glucose oxidase (CeO_2_-Au@GOx) as an electrochemical probe for signal amplification [52]. The biosensor provided an LOD of 4.3 × 10^−16^ M in the linear range from 1.0 × 10^−15^ to 1.0 × 10^−12^ M by DPV.

In a different report, an electrochemical biosensor for the simultaneous detection of miR-155 and miR-27b was described [76]. The strategy was based on the combination of the high base-mismatch selectivity of the ligase chain reaction (LCR), two reporting probes labeled with PbS and CdS QDs, and two capture probes co-immobilized onto the MBs. After the miRNA’s incubation with the modified capture and reporting probe conjugates, T4 DNA ligase was added, leading to the release of the disjoined PbS and CdS QDs barcodes from the MBs-conjugates. The miR-155 and miR-27b were detected by SWV, via the Pb and Cd oxidation peaks, in the linear ranges from 5.0 × 10^−14^ to 3.0 × 10^−11^ M for miR-155 and from 5.0 × 10^−14^ to 1.1 × 10^−9^ M for miR-27b, and with the LODs of 1.2 × 10^−14^ M miR-155 and 3.1 × 10^−14^ M miR-27b.

#### 2.1.4. Biosensors Based on Conducting Polymers

Several studies reported the development of electrochemical biosensors for miRNA detection based on the detection of the reversible oxidation of conjugated organic polymers, such as polypyrrole (PPy). The transduction consisted of measuring the modification of the electrochemical response of the conducting polymer after the hybridization of the miRNA target with the immobilized ssDNA probe.

miR-141, along with miR-103 and miR-29b-1 biomarkers for bladder and lung cancer, was detected by SWV at an electrochemical biosensor based on the bifunctional conducting polymer poly(5-hydroxy-1,4-naphthoquinone-co-5-hydroxy- 2-carboxyethyl-1,4-naphthoquinone) (poly(JUGco-JUGA)) acting both as immobilizing and transducing elements [60]. The GCE/poly(JUGco-JUGA) biosensor showed a linear range from 5.0 × 10^−13^ to 1.0 × 10^−10^ M and an LOD of 6.5 × 10^−13^ M.

A biosensor for the detection of miR-103, miR-141, and miR-29b-1 was also developed [49]. 5-hydroxy-3-hexanedithiol-1,4- naphthoquinone (JUG-SH) was used as the transducing element, while 6- mercaptohexanoic acid (6-MHA) was used for anchoring the NH_2_-ssDNAprobes, with both polymers self-assembled onto the surface of the Au NPs-modified GCE. The JUG-SH quinone groups were playing the role of the redox probe. The sensor quantitative performance was evaluated for miR-103 by SWV, showing a linear range from 1.0 × 10^−8^ to 5 × 10^−9^ M and an LOD of 1.0 × 10^−13^ M.

An electrochemical biosensor for the detection of miR-24 was built via the immobilization of ssDNA probes onto a GCE modified by a conducting polymer hydrogel formed by polyaniline (PANI) and phytic acid (PA) [61]. Using DPV, the sensor showed a linear range from 1.0 × 10^−15^ to 1.0 × 10^−12^ M and an LOD of 3.4 × 10^−16^ M.

### 2.2. Label-Free Electrochemical Biosensors

There are only very few reports concerning the label-free and reagentless electrochemical detection of microRNA breast cancer biomarkers.

Label-free electrochemical biosensors are advantageous over conventional bioassay techniques as they offer a rapid analytical response time, demand an ultra-low sample volume, and are easily integrated with modern gadgets useful for on-site utilities. However, achieving amplified sensitivity, reliability, and specificity without compromising on the inherent redox behavior is highly challenging.

#### 2.2.1. Biosensors Based on the Detection of Guanine Residues

**Figure 9:**
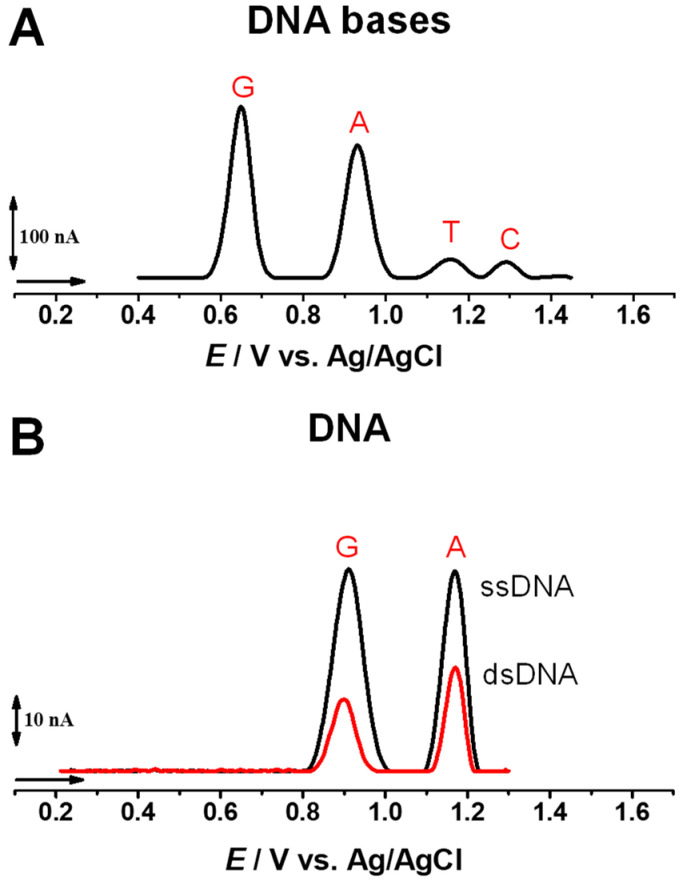
DPVs baseline corrected at GCE in solutions at pH 7.0: (**A**) 20 µM equimolar mixture of G, A, T, and C bases; and (**B**) 60 μg mL^−1^ dsDNA and 60 μg mL^−1^ ssDNA. Adapted from [8] with permission.

The DNA bases guanine (G), adenine (A), thymine (T), and cytosine (C) are electroactive and their oxidation at GCE follows the order of: *E*_p_^G^ = ~+0.70 V < *E*_p_^A^ = ~+0.96 V < *E*_p_^T^ = ~+1.16 V < *E*_p_^C^ = ~+1.31 V, vs. Ag/AgCl, 3 M KCl, at pH = 7.0 [102], as shown in Figure 9A. The oxidation of double-stranded DNA (dsDNA) and ssDNA at GCE generally presents two pH-dependent anodic peaks, corresponding to the oxidation of G and A residues oxidation, as shown in Figure 9B [6,8], while the T and C residue oxidation occurs at high positive potentials, near the potential of oxygen evolution, and are not usually detected.

A first report concerning the direct and label-free detection of miRNA was based on the detection of G residues’ electrochemical oxidation using DPV [78]. The signal-on detection of miR-122 was achieved by following the occurrence of the G residues’ oxidation peak of the miR-122 target after the hybridization with ssDNA capture probe immobilized onto SPGEs. The ssDNA capture probes presented the G residues substituted by inosine; therefore, only the target miR-122 contributed to the G oxidation peak current. The sensor showed an LOD of 5.0 × 10^−9^ M [78].

Furthermore, based on the DPV detection of the G residues’ oxidation peak, an electrochemical DNA biosensor for miR-34a was built by immobilizing the ssDNA probes onto GO-modified disposable PGEs [56]. The hybridization with the target miR-34a was followed at the GO-PGE/ssDNA biosensor in the linear range from 1.0 × 10^−7^ to 4.0 × 10^−2^ g L^−1^ with an LOD of 7.0 × 10^−7^ (5.0 × 10^−3^ g L^−1^). In a different report, the same DPV methodology for miR-34a detection employed an ionic liquid (IL)-modified chemically activated (CA) PGE, showing a linear range from 2.0 × 10^−3^ to 10 × 10^−2^ g L^−1^ and an LOD of 8.8 10^−4^ g L^−1^ (1.3 × 10^−7^ M) [80].

#### 2.2.2. Biosensors Based on Hemin/GQ DNAzymes

Hemin/G-quadruplex (GQ) DNAzyme electrochemical biosensors represent one of the most popular building assays of label-free electrochemical biosensors. In peroxidase hemin/GQ DNAzyme, the complex formed by hemin, an iron-containing porphyrin, with GQ DNA sequences leads to an improved peroxidase activity of hemin [103].

Based on this strategy, a TWJ DNA electrochemical biosensor for let-7 detection was developed [83]. The system contained three DNA hairpins and SH-ssDNA probes able to self-assemble into GQ structures, immobilized onto the gold electrode. The target let-7 triggered the formation of trivalent DNAzyme junctions, while the integration of CHA and DNAzyme amplifications enhanced the signal outputs. The sensor showed a linear range by DPV from 1.0 × 10^−15^ to 1.0 × 10^−9^ M and an LOD of 4.6 × 10^−16^ M.

**Figure 10:**
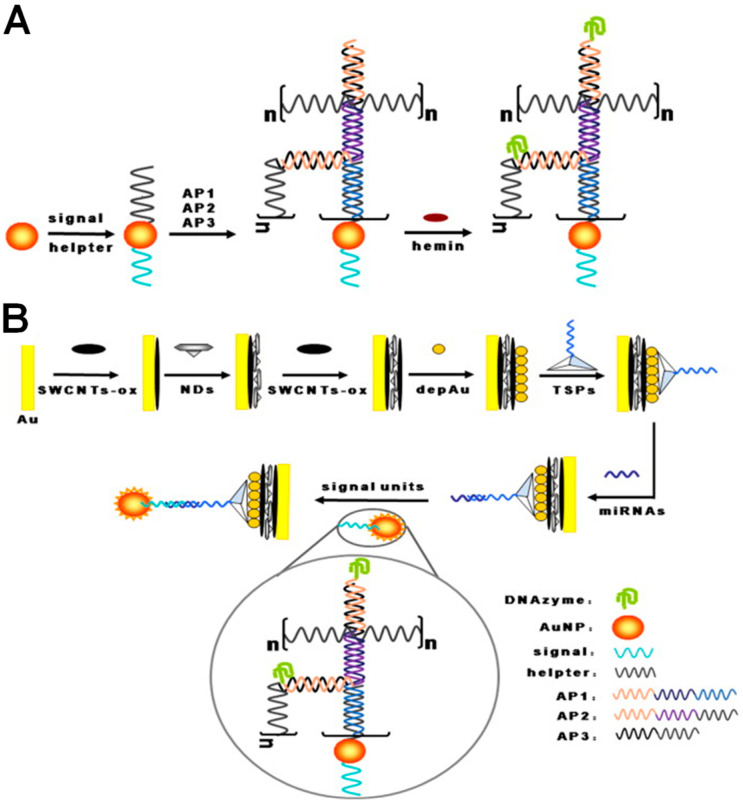
Schematic representation of the (**A**) DNA-functionalized Au NPs-based HCR and (**B**) hemin/GQ DNAzyme-based electrochemical biosensor for miR-21 detection, where n represents the number of cycles. Reproduced from [48] with permission.

An electrochemical biosensor for the miR-21 detection was also described, based on the electrode fabrication with layer-by-layer assembly of oxidized single-walled CNs (SWCTNs) and nanodiamonds (NDs), and an amplification strategy consisting of a hybridization chain reaction (HCR) and long hemin/GQ DNAzymes nanowires, as shown in Figure 10 [48]. The sensor presented a linear range from 1.0 × 10^−14^ to 1.0 × 10^−9^ M and an LOD of 2.0 × 10^−15^ M.

## 3. Electrochemical Biosensors for BRCA1 Analysis

All electrochemical strategies for the detection of BRCA1 were based on hybridization events between the BRCA1 target and the complementary DNA or PNA capture probe, as shown in Table 1.

Several electrochemical biosensors for BRCA1 detection were based on redox mediators. A polysaccharide-amplified method for the electrochemical detection of BRCA1 was reported [84], consisting of the immobilization of PNA sequences complementary to BRCA1 onto a gold electrode. The phosphate sites of the BRCA1 targets were modified by carboxyl groups-containing polysaccharide chains via phosphate-Zr(IV)-carboxylate crosslinking. Then, the polysaccharide chains were decorated with Fc labels via affinity coupling between the cis-diol site and the phenylboronic acid (PBA) group. Using this methodology, the electroanalytical determination of BRCA1 showed a linear range from 1.0 × 10^−14^ to 1.0 × 10^−8^ M and an LOD of 2.9 × 10^−15^ M.

In addition, at a GCE, a polypeptide (PEP)-doped poly(3,4-ethylenedioxythiophene) (PEDOT) nanocomposite with a 3D microporous network structure was used for the attachment of complementary ssDNA probes [85]. The GCE/PEDOT/PEP biosensor showed, in the presence of the Mb redox label, a linear range from 1.0 × 10^−14^ to 1.0 × 10^−9^ M and an LOD of 3.4 × 10^−15^ M for BRCA1 detection.

An electrochemical biosensor was described based on ssDNA immobilization via bifunctional cross-linker glutaraldehyde (GLu) onto a Au electrode modified by three different generations of Fc-cored PAMAMs [86]. The biosensor showed a wide linear range from 1.3 × 10^−9^ to 2.0 × 10^−8^ M and an LOD of 4.0 × 10^−10^ M.

A recyclable electrochemical sensing platform for the determination of BRCA DNA was developed, based on the DNA hybridization and host-guest interaction [87]. In the presence of the target BRCA, HRP-labeled DNA/Au nanospheres (NSs) concatemers were linked to Fc-labeled DNA/Au NSs. The hybridized complex was further captured on the GCE modified by GO and cucurbit[7]uril (CB [7]) through the host-guest interaction between CB [7] and Fc, which brought the HRP label next to the electrode surface. The dual signal amplification using Fc and HRP NSs concatemers led to BRCA detection with an LOD of 2.5 × 10^−11^ M in the linear range from 1.0 × 10^−7^ to 5.0 × 10^−11^ M. The proposed detection strategy showed a good applicability in human serum samples.

An electrochemical biosensor for BRCA1 was prepared by modifying the GCE using a nanocomposite consisting of poly(dopamine-beta cyclodextrine-Cetyl trimethylammonium bromide doped with Ag NPs (P[DA-β-CD/CTAB])-Ag NPs) and functionalized mesoporous silica (mobile crystalline material-41 grafted by sulfonic acid, MCM-41-SO_3_H) [88]. To further amplify the electrochemical signal, the MCM-41-SO_3_H provided a suitable pore volume and functional groups to capture the HPR-labeled antibodies. The proposed sensor showed: (i) by DPV, a linear range from 1.6 × 10^−11^ to 1.0 × 10^−8^ g L^−1^; and (ii) by SWV, a linear range from 6.3 × 10^−10^ to 2.0 × 10^−9^ g L^−1^ and a limit of quantification (LOQ) of 3.0 × 10^−11^ g L^−1^.

In a different report, a double signal amplification strategy for the detection of miRNA of BRCA1 was developed based on a cyclic enzymatic signal amplification (CESA) with a double-strand specific nuclease (DSN) and triple-CdTe QD-labeled DNA nanocomposites (3-QD@DNA NC) as a cascade signal probe [89]. CESA was first used to recognize the target miRNA of BRCA1, then the DNA of the RNA-DNA duplex was selectively cleaved, and the free miRNA was released to trigger the second cycle. The biosensor exhibited a linear range from 5.0 × 10^−18^ to 5.0 × 10^−15^ M and an LOD of 1.2 × 10^−18^ M.

An electrochemical biosensor based on thiol-modified tetrahedral-DNA (SH-TDNA) probes and poly-adenine (polyA)-mediated Au NPs with immobilized ssDNA showed, after hybridization with the target BRCA1, a linear range from 1.0 × 10^−15^ to 1.0 × 10^−9^ M and an LOD of 1.0 × 10^−16^ M [90].

Several reports showed the development of label-free impedimetric biosensors for BRCA1. A label-free genosensor was developed by immobilizing the ssDNA probes at the surface of the GCE modified by 1-pyrenebutyric acid-N-hydroxysuccinimide ester (PANHS), MWCNTs, and rGO [91]. The biosensor showed: (i) for the GCE/PANHS/ssDNA configuration, a linear range from 1.0 × 10^−16^ to 1.0 × 10^−10^ mol L^−1^ and an LOD of 3.7 × 10^−17^ mol L^−1^; (ii) for the GCE/MWCNTs/PANHS/ssDNA configuration, a linear range from 1.0 × 10^−17^ to 1.0 × 10^−10^ mol L^−1^ and an LOD of 3.1 × 10^−18^ mol L^−1^; and (iii) for the GCE/rGO/MWCNTs/PANHS/ssDNA configuration, a linear range from 1.0 × 10^−18^ to 1.0 × 10^−10^ mol L^−1^ and an LOD of 3.5 × 10^−19^ mol L^−1^.

Also at a GCE, complementary BRCA1 ssDNA sequences were immobilized after the modification of the electrode surface with a cross-linked polyethylene glycol (PEG) film containing amine groups, followed by the self-assembly of Au NPs [92]. The impedimetric label-free sensor showed a linear range from 5.0 × 10^−14^ M to 1.0 × 10^−9^ M and an LOD of 1.7 × 10^−15^ M.

**Figure 11:**
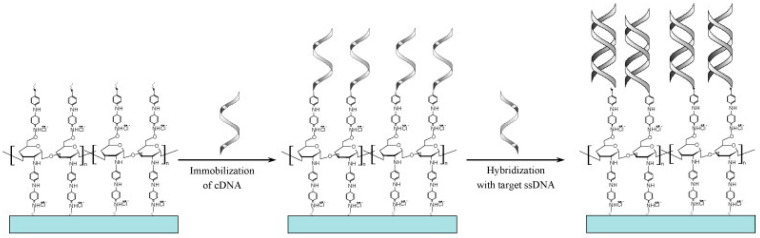
Schematic representation of the fabrication and detection strategy of an ITO/CHIT-co-PANI/ssDNA biosensor for BRCA1 detection. Reproduced from [93] with permission.

An impedimetric electrochemical biosensor based on chitosan-co-PANI (CHIT-co-PANI) copolymer coated onto indium-tin-oxide (ITO) was used for the hybridization of immobilized complementary ssDNA probes with the BRCA1 target, as shown in Figure 11 [93]. The biosensor showed a linear range from 5.0 × 10^−17^ to 2.5 × 10^− 14^ M and an LOD of 5.0 × 10^− 17^ M [93].

In another report, a label-free EIS method for BRCA1 detection, based on the immobilization of SH-ssDNA onto a Au electrode and its further hybridization with a complementary BRCA1 target, was developed [94]. The genosensor showed a linear range from 1.0 × 10^−19^ to 1.0 × 10^−7^ M and an LOD of 4.6 × 10^−20^ M.

An impedimetric biosensor platform was also developed by immobilizing the SH-ssDNA probes onto a GCE modified by PEG grafted on a Fe(III)-tannic acid (TA)/poly-dopamine (pDA) coating via a layer-by-layer technique [95]. In the presence of Au NPs, the sensor showed a linear range from 1.0 × 10^−16^ to 1.0 × 10^−11^ M and an LOD of 5.0 × 10^−17^ M.

## 4. Conclusions

Breast cancer is associated with a high incidence rate, recurrence, and metastasis; therefore, it remains the second leading cause of cancer mortality in woman. Conventional strategies of breast cancer biomarkers’ detection are expensive, laborious, time-consuming, and require highly trained biologists.

This review presents a comprehensive overview concerning the design, development, and applications of electroanalytical methods of detection of nucleic acid breast cancer biomarkers, particularly miRNAs and BRCA1. The electrochemical biosensors fabrication approaches, the signal amplification approaches, the detection techniques, and the key performance parameters, such as the linearity range and limit of detection, were presented.

DNA electrochemical biosensors are considered extremely advantageous devices for evaluating the levels of nucleic acid breast cancer biomarkers due to their high sensitivity and selectivity, cost-effectiveness, short procedure time, simplicity, ability to be miniaturized, and high potential applicability in urine, blood, and tissue samples.

The challenge in the development of electrochemical biosensors for nucleic acid breast cancer biomarkers is to achieve the electrochemical transduction associated with the hybridization events that do not actually involve charge transfer reactions. Nanostructured materials, such as carbon nanotubes, graphene, graphene oxide, reduced graphene oxide, graphene quantum dots, metal nanoparticles and nanostructures, magnetic beads, polymers, hydrogels, dendrimers, and nanocomposites, are playing an increasingly critical role in their fabrication and are expected to be used more in the future. Generally, they represent the most attractive approach because they not only allow the amplification of the electrochemical signal and the improvement of the biosensor sensitivity, but they can also act as signal tags or signal reporters and can form highly conductive nanostructured electrochemical platforms.

Nevertheless, further studies are needed to enhance the biosensors’ stability and reproducibility, especially in biological matrixes, avoiding misleading results due to DNA and/or RNA sequences with similar structures and interferents. Moreover, challenges in the miniaturization of the biosensor devices and the integration of microfluidics technologies still need to be solved in order to develop commercially available point-of-care devices.

## Data Availability

Not applicable.
